# Analysis of the Shear Behavior of Stubby Y-Type Perfobond Rib Shear Connectors for a Composite Frame Structure

**DOI:** 10.3390/ma10111340

**Published:** 2017-11-22

**Authors:** Sang-Hyo Kim, Kun-Soo Kim, Do-Hoon Lee, Jun-Seung Park, Oneil Han

**Affiliations:** School of Civil & Environmental Engineering, Yonsei University, Yonsei-Ro 50, Seodaemun-Gu, Seoul 03722, Korea; sanghyo@yonsei.ac.kr (S.-H.K.); kun-soo_kim@yonsei.ac.kr (K.-S.K.); dohoonlee@yonsei.ac.kr (D.-H.L.); junseungpark@yonsei.ac.kr (J.-S.P.)

**Keywords:** stubby Y-type perfobond rib shear connectors, composite frame structure, shear strength, ductility, push-out test

## Abstract

Shear connectors are used in steel beam–concrete slabs of composite frame and bridge structures to transfer shear force according to design loads. The existing Y-type perfobond rib shear connectors are designed for girder slabs of composite bridges. Therefore, the rib and transverse rebars of the conventional Y-type perfobond rib shear connectors are extremely large for the composite frames of building structures. Thus, this paper proposes stubby Y-type perfobond rib shear connectors, redefining the existing connectors, for composite frames of building structures; these were used to perform push-out tests. These shear connectors have relatively small ribs compared to the conventional Y-type perfobond rib shear connectors. To confirm the shear resistance of these stubby shear connectors, we performed an experiment by using transverse rebars D13 and D16. The results indicate that these shear connectors have suitable shear strength and ductility for application in composite frame structures. The shear strengths obtained using D13 and D16 were not significantly different. However, the ductility of the shear connectors with D16 was 45.1% higher than that of the shear connectors with D13.

## 1. Introduction

Steel–concrete composite structural systems with shear connectors have excellent structural performance and economic feasibility, and have been employed in various fields for decades. In particular, beam–slab composite systems have been widely used in building and bridge structures, and are beneficial for withstanding extreme conditions such as fire and earthquake. Kodur et al. [1,2] studied the behavior of composite steel girders during a fire through experiments and analyses, while Kim et al. [3], Bursi et al. [4], and Nakashima et al. [5,6] experimentally analyzed composite structures under cyclic loading. The shear resistance secured by the shear connectors is determined using the design shear force. In addition, shear stiffness determines the degree of shear connection, and ductility prevents brittle failure of the shear connectors. The behaviors of composite beams with respect to shear connectors have been investigated by numerous researchers. Kim and Jeong [7] conducted an experimental study to verify the ultimate behavior of a composite deck system with respect to steel sheets and perfobond rib shear connectors. They performed beam and push-out tests of the shear connectors and composite beams, and verified their load-carrying capacity. Qureshi et al. [8] developed a three-dimensional nonlinear numerical model for a composite beam using profiled sheeting and stud shear connectors, and used the model to obtain the shear strength, relative slip, and failure modes. Vasdravellis and Uy [9] performed an experimental and numerical study on the shear capacity and moment–shear interaction of composite beams. They aimed to assess the contribution of the concrete slab to the ultimate shear capacity of a composite section and develop a moment–shear interaction law for composite beams subjected to combined positive bending and shear. They found that a higher degree of shear connection increases the shear capacity of a composite beam, and the level of increase is higher in beams with a larger slab-depth slenderness ratio (depth of slab/depth of beam). In addition, the contribution of the slab is a linear function of the slab-depth slenderness ratio of the composite section. Shariati et al. [10] conducted push-out tests of the channel and angle shear connectors in high-strength concrete to compare their shear strengths. The channel shear connectors were found to have higher ductility than the angle shear connectors, and higher performance due to the higher height of the connectors. Lasheen et al. [11] compared the behavior of lightweight and normal weight concretes in eight composite beams with channel shear connectors. The findings showed that the lightweight concrete only slightly affected the load capacity in comparison to the normal weight concrete.

Studies on composite structures were first conducted in the 1920s. Caughey [12] stressed the need for shear connectors that can resist horizontal shear force. The stud shear connector, which is commonly utilized in steel–concrete composite systems, has been studied for many years. In 1956, Viest [13] performed a static load test by using a stud connector to propose an equation for shear strength, and modified this equation in the 1960s [14]. Subsequently, the shear strength of stud shear connectors was studied by considering various variables, such as the cross-section, height, and tensile strength of the stud, as well as the elastic modulus and compressive strength of the concrete [15,16,17]. Large stud shear connectors greater than 22 mm in diameter have also been studied [18,19,20]. At a German design company, Leonhardt and Zellner [21] developed a new type of a shear connector—that is, the perfobond rib shear connector—to solve the fatigue problem of stud shear connectors. Oguejiofor and Hosain [22,23,24] compared the behaviors of the perfobond rib shear and stud connectors by analyzing the differences in their failure modes in push-out and beam tests. They then proposed an equation for evaluating the strength of the perfobond rib shear connector by considering the tensile strength of concrete, amount of transverse rebar, and location of holes. Valente and Cruz [25] conducted an experimental analysis to compare shear behaviors, such as shear strength and ductility, of various connector types and conducted push-out tests for three types of shear connectors: stud, perfobond, and T-connector. Vianna et al. [26,27,28] conducted a push-out test and numerical analysis on the T-type shear connector in a composite beam girder. The results displayed that the performance of the T-perfobond connector and shear resistance were affected by the thickness of the concrete slab. Lorenc et al. [29,30] performed an experimental study and a numerical analysis on composite dowels with puzzle-like shapes. Papastergiou et al. [31] proposed a new type of shear connector using friction and bond effects, and identified its behavior through experimental analysis. The Y-type perfobond shear connector developed based on various types of shear connectors was observed to have outstanding shear resistance and ductility [32], and exhibited good structural performance under the cyclic design load of bridges [33]. To predict the shear strength of Y-type perfobond shear connectors, Kim et al. [34,35,36] conducted push-out tests, beam tests, and numerical analysis and proposed shear resistance formulas by considering design variables.

In building structures, the shear force exerted on the composite frame by design loads is smaller than that on composite bridges. The existing Y-type perfobond rib shear connectors [32,33,34,35,36] are designed for the girder slabs of composite bridges. Therefore, the rib and transverse rebars of the conventional Y-type perfobond rib shear connectors are extremely large for the composite frames of building structures. This conventional connector has a rib height and width 100 and 80 mm respectively, and the spacing of the dowel hole is 120 mm. However, the slabs of building structures have a smaller thickness than those of composite bridges, and the spacings of reinforcements for building structures are narrow. In addition, the diameter of transverse rebars used in composite buildings is smaller than that of composite bridges. Thus, the dimension of the shear connector should be modified to the stubby Y-type perfobond rib shear connector. To use Y-type perfobond rib shear connectors in composite frame structures, various design factors, such as the compressive strength of concrete, the height of the slab, and the diameter of the transverse rebar, must be considered. To this end, the current study proposed the stubby Y-type perfobond rib shear connectors for composite frames, and experimentally examined their shear strength and ductility through push-out tests. All the dimensions of the specimens were determined considering the concrete slab, and then the shear resistance, ductility, and fracture modes were confirmed at the shear connection area compared with the shear resistance equation.

## 2. Push-Out Tests of a Composite Structure Using Stubby Y-Type Perfobond Rib Shear Connectors

### 2.1. Test Specimens

**Figure 1:**
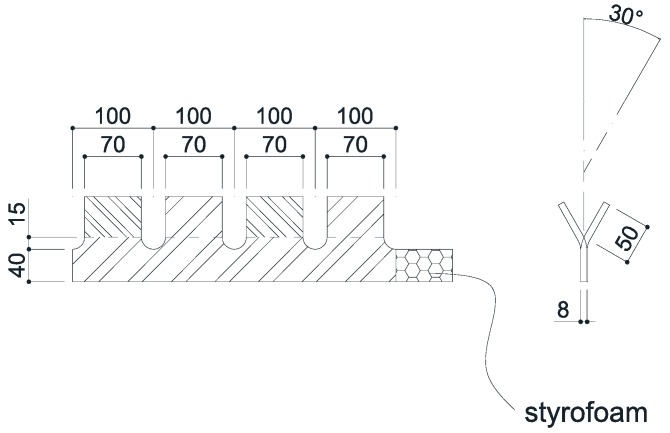
Dimensions of Y-type perfobond rib shear connector (unit: mm).

**Figure 2:**
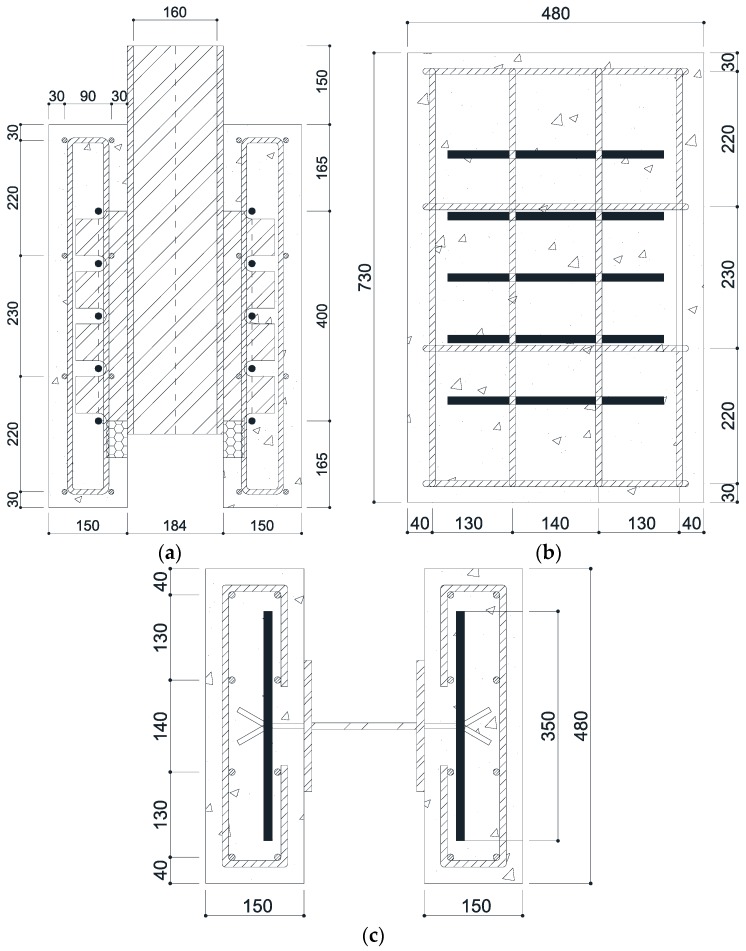
Dimensions of push-out test specimen (unit: mm). (**a**) Front view; (**b**) Side view; (**c**) Plan view.

The targeted compressive concrete strength of the stubby Y-type perfobond rib shear connector was limited to 27 MPa, while the ultimate strength of a stubby Y-type perfobond rib shear connector with 4 ribs was limited to approximately 450 kN. The push-out test specimens were manufactured according to the standard of a direct shear specimen suggested in the Eurocode-4 guidelines [37]. The main design variables are the width and height of the rib, and the diameters of the dowel hole and transverse rebar. As the shear force recommended for a building structure is smaller than that of a bridge structure, Kim et al. [32] suggested a smaller-sized Y-type perfobond rib shear connector than the existing connector. As shown in Figure 1, the shear connector had a Y-shaped angle of 60°, rib height of 50 mm, width of 70 mm, thickness of 8 mm, hole diameter of 30 mm, and transverse rebar diameter of 13 mm (D13) or 16 mm (D16). The shear connector specimens were classified into two types: SY-D13-M and SY-D16-M, based on the transverse rebar diameters. The concrete block of the specimens was determined to have a thickness, width, and length of 150, 480, and 730 mm, respectively. The slab of the push-out specimens were designed by considering the concrete thickness generally used for building structures. Hence, concrete with a designed compressive strength of 27 MPa was utilized. Twelve concrete cylindrical specimens and six push-out test specimens were cured using the steam curing method. Each group contained three cylindrical test specimens, and was tested at curing periods of 21 and 28 days and on the day of the push-out test. Table 1 presents the compressive test results for the concrete specimens. The tensile strength tests of structural steel for the stubby Y-type perfobond ribs were conducted using the push-out test specimens. The tensile strength, yield strength, and ductility were obtained through these test results. Table 2 provides the results of the tensile strength tests. A rib height of 50 mm was designed in consideration of a concrete slab height of 150 mm, which is commonly used for building structures. A rib width of 70 mm was designed in consideration of a distance of 100 mm between the transverse rebars. Grease was applied to the rib before pouring the concrete in order to eliminate the adhesive force caused by the chemical bonding between the concrete and rib. A 70-mm-long piece of styrofoam was installed at the bottom end in the opposite direction of the applied load of the rib to prevent concrete bearing resistance any part other than on the Y-shape and dowel hole. Figure 2 shows the dimensions of the push-out test specimens used for testing the stubby Y-type perfobond rib shear connectors, and Table 3 lists the specifications of the stubby shear connectors.

### 2.2. Test Procedure

**Figure 3:**
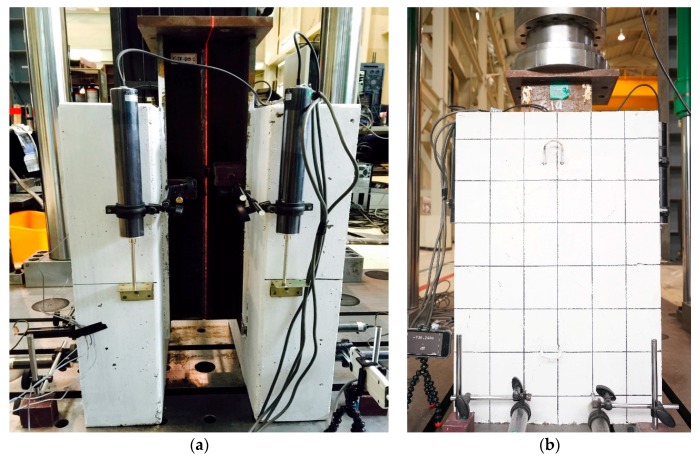
Push-out test setup: (**a**) Front view; (**b**) Side view.

The push-out test of the stubby Y-type perfobond rib shear connectors was conducted using a 1000 kN universal testing machine (Samyeon Tech., Seismic Simulation Test Center in Pusan National University, Busan, Korea). The relative displacement between the concrete and steel was measured using four linear variable differential transducers (LVDTs, Tokyo Sokki Kenkyujo Co., Ltd., Seismic Simulation Test Center in Pusan National University, Busan, Korea) attached to L-shaped aluminum angles. The LVDTs were installed 365 mm below the top of the concrete slab. Grid lines were drawn on the concrete surface of all the specimens, and high-resolution camera was used to record the cracks. A monotonic load was applied in the displacement a control mode, and the load rate was set to 0.02 mm/s to prevent failure within 15 min, according to Eurocode-4 [37]. Figure 3 shows the setup of the push-out test, which was stopped when the load decreased to less than 80% of the ultimate load; Table 4 shows the list of push-out tests. To confirm the deformation of the transverse rebars and stubby ribs for each load step in SY-D13-M1 and SY-D16-M1, the push-out tests were terminated at displacements where the load was 80% of the shear strength. For SY-D13-M2 and SY-D16-M2, the tests were terminated at displacements where the stiffness was recovered. To confirm sufficient deformation of the transverse rebar and rib in SY-D13-M3 and SY-D16-M3, the load was applied until the point at which the displacement was 25 mm. After the push-out tests, the concrete blocks of the specimens were crushed to confirm the deformation of the transverse rebars and stubby Y-type perfobond ribs.

## 3. Shear Strength and Ductility of Composite Structures using Stubby Y-Type Perfobond Rib Shear Connectors

**Figure 4:**
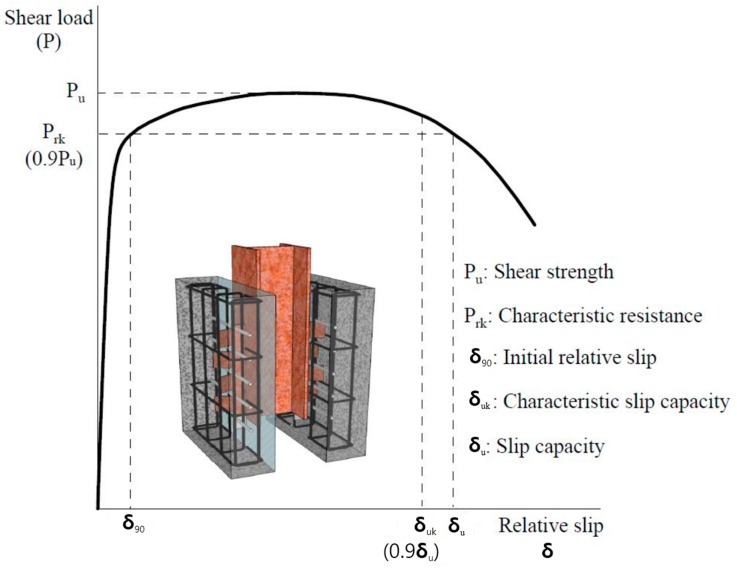
Determination of shear capacity and relative slip.

The test objective was to analyze the change in the shear force according to the diameter of the transverse rebar for which the dimensions of the stubby Y-type perfobond rib shear connectors were fixed. This is because the stubby Y-type perfobond rib shear connector can be applied to various sizes of a transverse rebar used in composite building structures. To compare the shear strength and ductility based on push-out tests, the shear strength (P_u_), characteristic resistance (P_rk_), initial relative slip (δ_90_), characteristic slip capacity (δ_uk_), and slip capacity (δ_u_) were defined, as shown in Figure 4 [32]. Eurocode-4 [37] defines a shear connector as ductile if δ_uk_ > 6 mm. In addition, Kim et al. [32] suggested using the ratio of the slip capacity and initial relative slip (δ_u_/δ_90_) to estimate the ductility in the inelastic behavior region of a shear connector by considering initial stiffness. Moreover, Kim et al. [35] proposed Equation (1) to predict the shear strength of a Y-type perfobond rib shear connector. Table 5 compares the tested and predicted shear strengths of SY-D13-M and SY-D16-M.

(1) $$Q=3.372\times \left(\frac{d}{2}+2h\right)t\times f_{ck}+1.213\times r\times A_{tr}\times f_{y}+1.9\times n\times \pi \times {\left(\frac{d}{2}\right)}^{2}\times \sqrt{f_{ck}}+0.757\times m\times h\times s\times \sqrt{f_{ck}},$$

where Q represents the shear resistance (kN), d is the diameter of the dowel hole (mm), h is the individual rib height (mm), t is the rib thickness (mm), $f_{ck}$ is the compressive strength of the concrete (MPa), r is the number of transverse rebars, $A_{tr}$ is the cross-sectional area of the transverse rebar (mm^2^), $f_{y}$ is the yield strength of the transverse rebar (MPa), n is the number of dowel holes, m is the number of dowel areas formed between the ribs bent in a Y-shape, and s is the net distance between the ribs bent in the same direction (mm).

**Figure 5:**
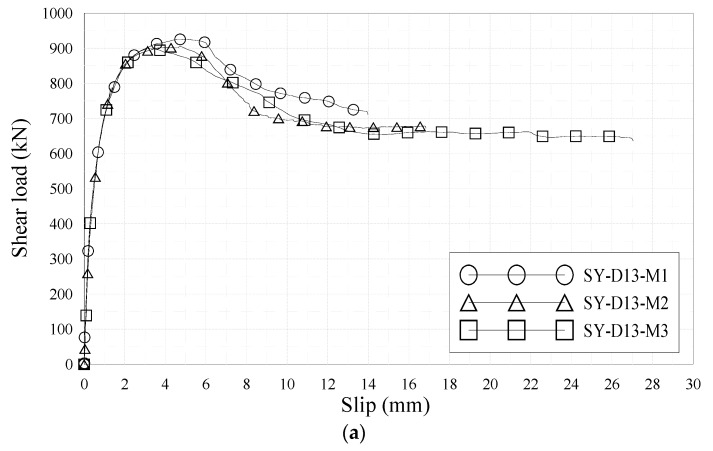

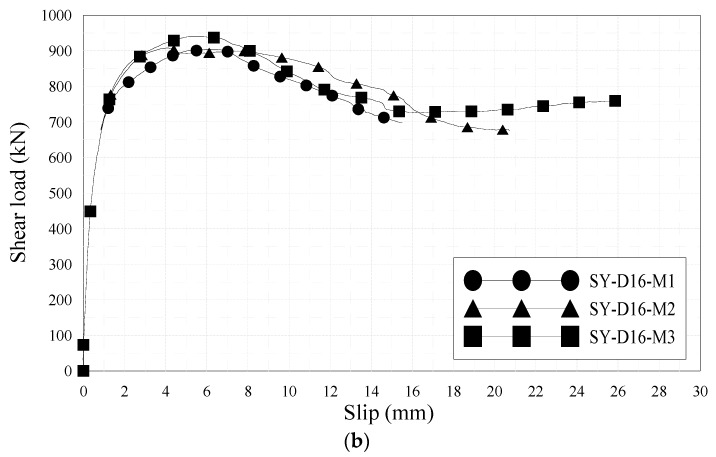
Load–slip relationships: (**a**) SY-D13-M; (**b**) SY-D16-M.

Figure 5 and Table 5 present the push-out test results. In the cases of SY-D13-M1/M2/M3, the shear strengths obtained were 925.2, 904.4, and 898.7 kN, respectively, and the average shear strength was 897.3 kN. The ductilities calculated according to Eurocode-4 [37] 6.90 mm and the result obtained by the evaluation formula (δ_u_/δ_90_) suggested by Kim et al. [32] was 4.82. In the cases of SY-D16-M1/M2/M3, the shear strengths obtained were 904.1, 907.7, and 939.7 kN, respectively, with an average of 912.17 kN. Moreover, the ductilities calculated according to Eurocode-4 [37] were 10.01 and the result of the evaluation formula (δ_u_/δ_90_) suggested by Kim et al. [32] was 6.21.

The difference between the shear strengths of SY-D13-M and SY-D16-M was 12.8 kN, with SY-D16-M exhibiting 1.4% higher shear strength. Based on these results, the effect of the change in shear strength due to the rebar sizes of D13 and D16 is not much. However, the load reduction is greater for SY-D13-M than for SY-D16-M, both of which satisfied the ductility standard for shear connectors defined by Eurocode-4 [37]. The δ_uk_ of SY-D13-M was 6.90 mm, which slightly exceeds the ductility standard suggested by Eurocode-4 [37], while that of SY-D16-M was 10.01 mm, which significantly exceeds the same standard. When evaluating ductility based on the initial stiffness, δ_u_, δ_90_, and δ_u_/δ_90_ of SY-D13-M were 7.67 mm, 1.59 mm, and 4.82, respectively, while those of SY-D16-M were 11.12 mm, 1.79 mm, and 6.21, respectively. The difference between the δ_90_ values of SY-D13-M and SY-D16-M was 0.02 mm (11% for δ_90_ of SY-D16-M), and the difference between their δ_u_ values was 36.45 mm (31% for δ_u_ of SY-D16-M). That is, larger-diameter transverse rebars show more ductility after yield strength than the initial shear behavior. Based on both ductility evaluation methods, shear connectors with large-diameter rebars are preferable in terms of ductility.

The variables applied to Equation (1) in [32] to evaluate the shear resistance are listed in Table 6. As a result, the shear strengths of SY-D13-M and SY-D16-M predicted using Equation (1) in [32] were 803.5 and 1082.6 kN, and the experimental results were 894.6 and 907.4 kN, respectively. In the case of SY-D13-M, the average shear strength estimated in the push-out tests was 1.1 times the shear strength estimated using the equation. Moreover, the average shear strength of SY-D16-M in the push-out tests was 0.84 times the shear strength estimated using the equation. In other words, the measured shear strength of SY-D13-M was greater than the predicted shear strength, while that of SY-D16-M was lower than the predicted shear strength. As the difference between the measured and predicted strengths was approximately 13%, the shear strength equation for Y-type perfobond rib shear connectors can also be applied to stubby Y-type perfobond rib shear connectors. However, the influence of the transverse rebar was found to be overestimated.

## 4. Failure of Composite Structures Using Stubby Y-Type Perfobond Rib Shear Connectors

### 4.1. Concrete Crack Patterns and Failure of Stubby Y-Type Perfobond Rib Shear Connectors

**Figure 6:**
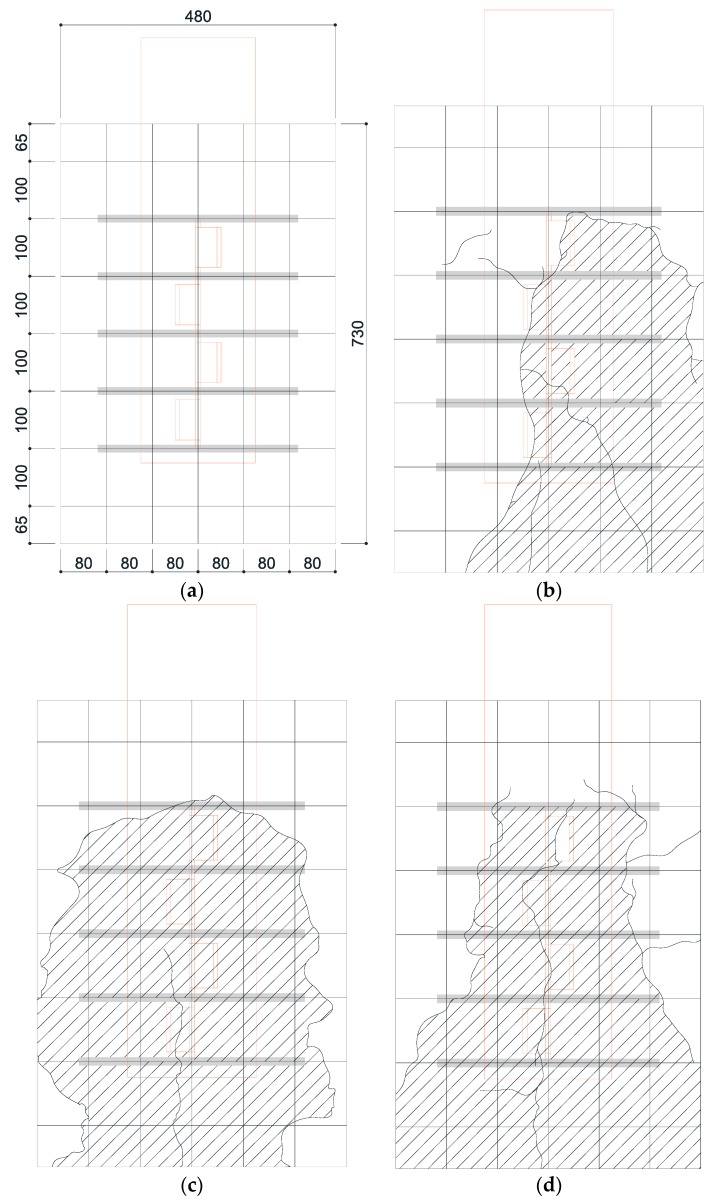
Concrete crack patterns of SY-D13: (**a**) Grid lines (Unit: mm); (**b**) M1 (Slashed area: Pry-out failure area); (**c**) M2 (Slashed area: Pry-out failure area); (**d**) M3 (Slashed area: Pry-out failure area).

**Figure 7:**
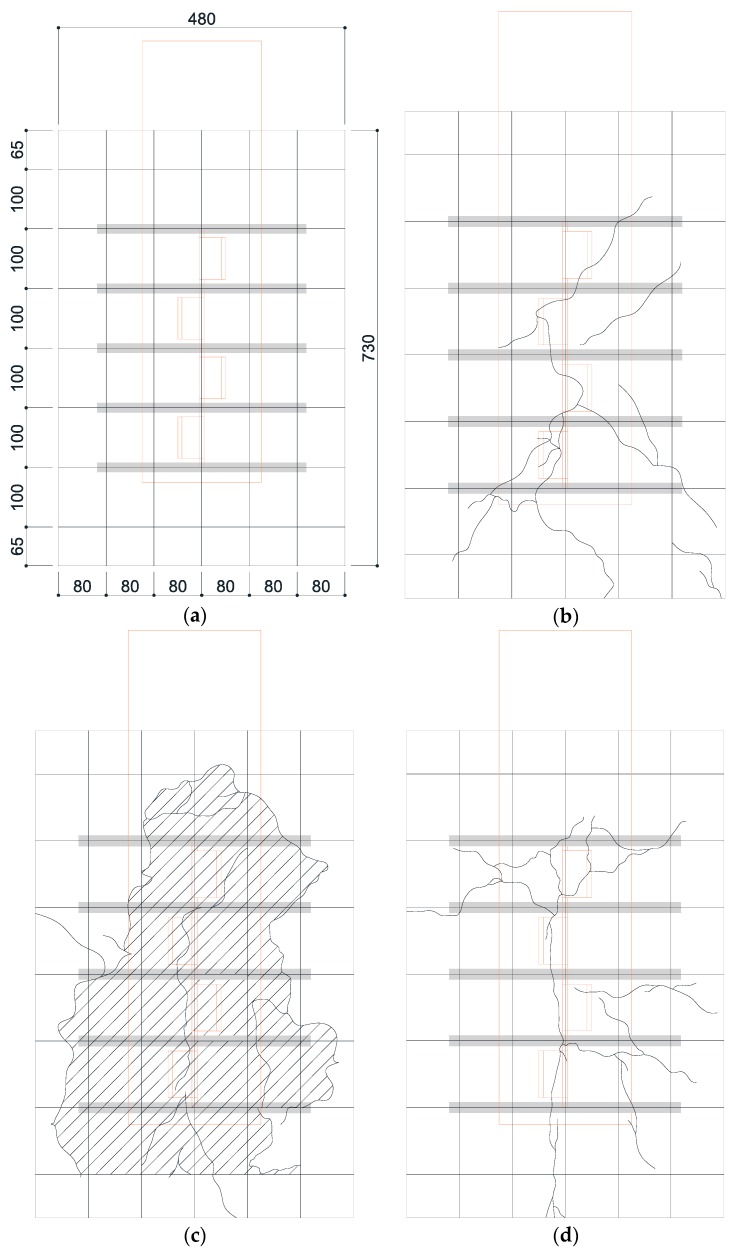
Concrete crack patterns of SY-D16: (**a**) Grid lines (Unit: mm); (**b**) M1; (**c**) M2 (Slashed area: Pry-out failure); (**d**) M3.

**Figure 8:**
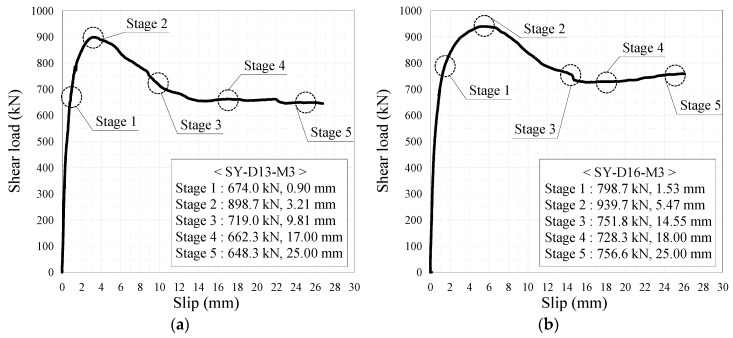
Loading stage: (**a**) SY-D13-M3; (**b**) SY-D16-M3.

As mentioned earlier, the crack occurrence and propagation on concrete surfaces were recorded using a high-resolution camera. Figure 6 and Figure 7 show the crack patterns of SY-D13-M and SY-D16-M, respectively, after the push-out tests. Both specimens exhibited similar crack patterns. In SY-D13-M and SY-D16-M2, the pry-out failure of concrete occurred as shown in the shaded areas of Figure 6 and Figure 7, respectively. However, SY-D16-M1 and SY-D16-M3 were destroyed because of the splitting failure of the concrete slab. To gradationally confirm the crack distribution, the crack distributions of SY-D13-M3 and SY-D16-M3 with the largest deformation were divided into the following five stages (Figure 8):

- Stage 1: Occurrence of initial cracks (SY-D13-M3: 75% P_u_; SY-D16-M3: 85% P_u_)
- Stage 2: Shear strength (P_u_)
- Stage 3: 80% shear strength
- Stage 4: Stiffness recovery (SY-D13-M3, δ = 17 mm; SY-D16-M3, δ = 18 mm)
- Stage 5: Ultimate limit state (δ = 25 mm)

**Figure 9:**
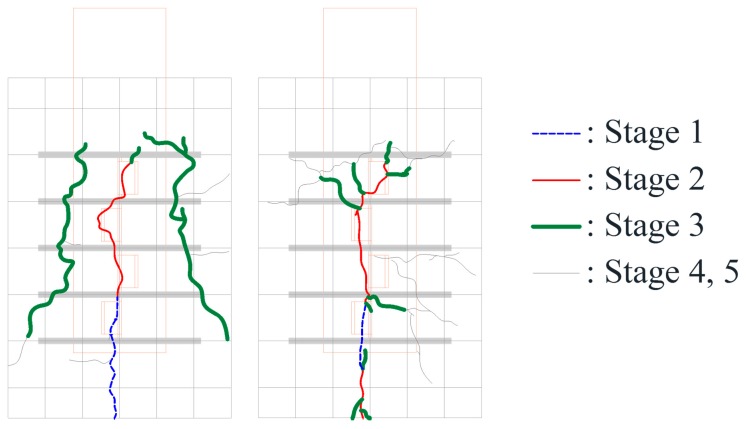
Crack pattern in each stage: (**a**) SY-D13-M3; (**b**) SY-D16-M3.

Figure 9 and Table 7 show the crack distribution in each stage. In the case of SY-D13-M3 (Figure 9), the crack in Stage 1 initiated as a splitting crack from the bottom end of the cut rib and progressed upward in the specimen. In Stage 2, the splitting crack progressed in the vertical direction, along the center of the rib. In Stage 3, additional splitting cracks occurred toward both the sides of the rib, and further progressed in the vertical direction. Stages 4 and 5 displayed the occurrences of even more cracks from the cracks developed in the previous stages in the lateral direction along the outer perimeter of the concrete slab. Finally, failure of concrete occurred as pry-out failure near the upper rib. In the case of SY-D16-M3, Stage 1 initiated as a splitting crack from the bottom end of the rib, as in SY-D13-M3. In Stage 2, the crack progressed in the vertical direction along the center, and in Stage 3, this crack progressed in the horizontal direction along the section arranged with the transverse rebar. In Stages 4 and 5, these horizontal cracks progressed further, and a new horizontal crack occurred. Unlike in the case of SY-D13-M3, the failure in SY-D16-M3 was not a pry-out failure but a splitting failure of the concrete slab.

Both SY-D13-M3 and SY-D16-M3 exhibited initial cracks along the vertical direction from the bottom end of the rib in Stages 1 and 2. However, from Stage 3, they exhibited different behaviors. SY-D13-M3 exhibited a crack in the vertical direction that continued from approximately the center of the rib, while SY-D16-M3 exhibited a crack that progressed along the horizontal direction from the direction in which the transverse rebar was arranged. Finally, SY-D13-M3 showed a pry-out failure of concrete, while SY-D16-M3 showed a splitting failure of concrete. It was assumed that, in the case of SY-D13-M3, which has a relatively small transverse rebar cross-section, the pry-out failure resulted from the local damage of the concrete near the rib. In the case of SY-D16-M3, as the bonded surface area and cross-sectional area of D16 transverse rebar were larger than those of D13, the horizontal strain of D16 transverse rebar occurred lower than that of D13. Thus, the deformation of the D16 transverse rebar was relatively small, and the load was evenly dispersed over the entire concrete slab owing to its relatively large cross-section. Therefore, the horizontal crack in the D16 transverse rebar occurred largely around it, leading to a splitting failure, compared to that in the D13 transverse rebar.

### 4.2. Deformation of Ribs and Transverse Rebars of Stubby Y-Type Perfobond Rib Shear Connectors

**Figure 10:**
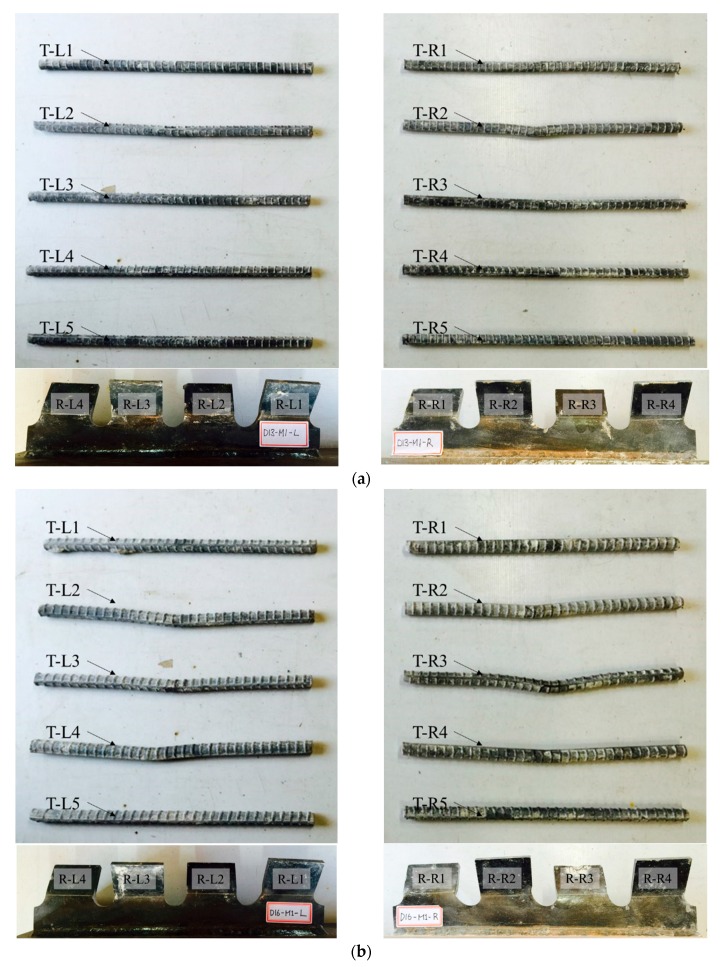
Deformation of transverse rebars and ribs; Stage 3: (**a**) SY-D13-M1; (**b**) SY-D16-M1.

**Figure 11:**
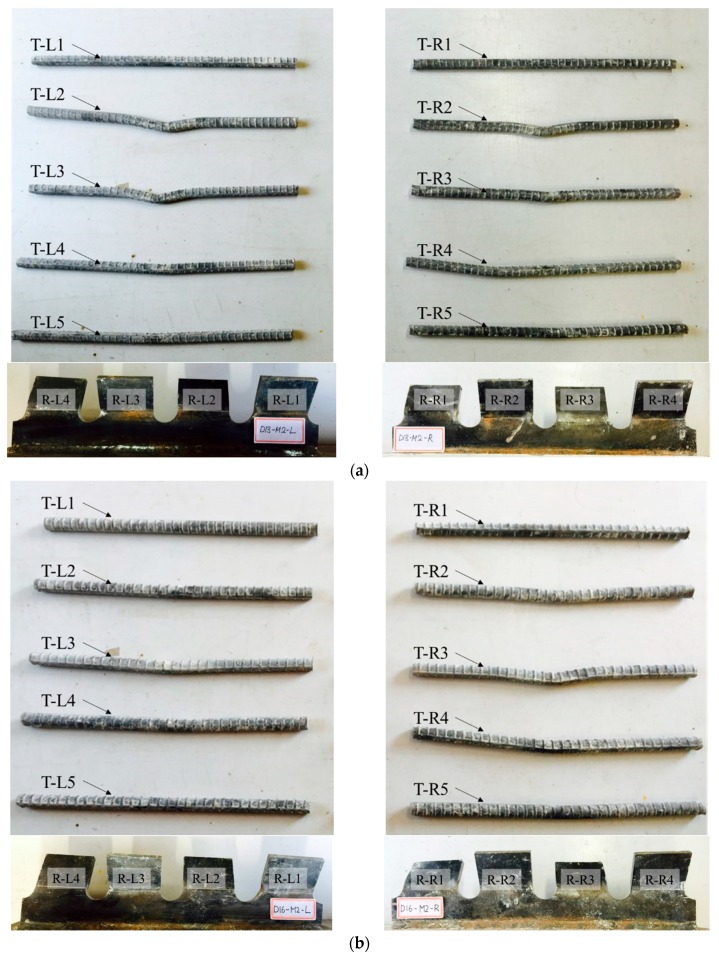
Deformation of transverse rebars and ribs; Stage 4: (**a**) SY-D13-M2; (**b**) SY-D16-M2.

**Figure 12:**
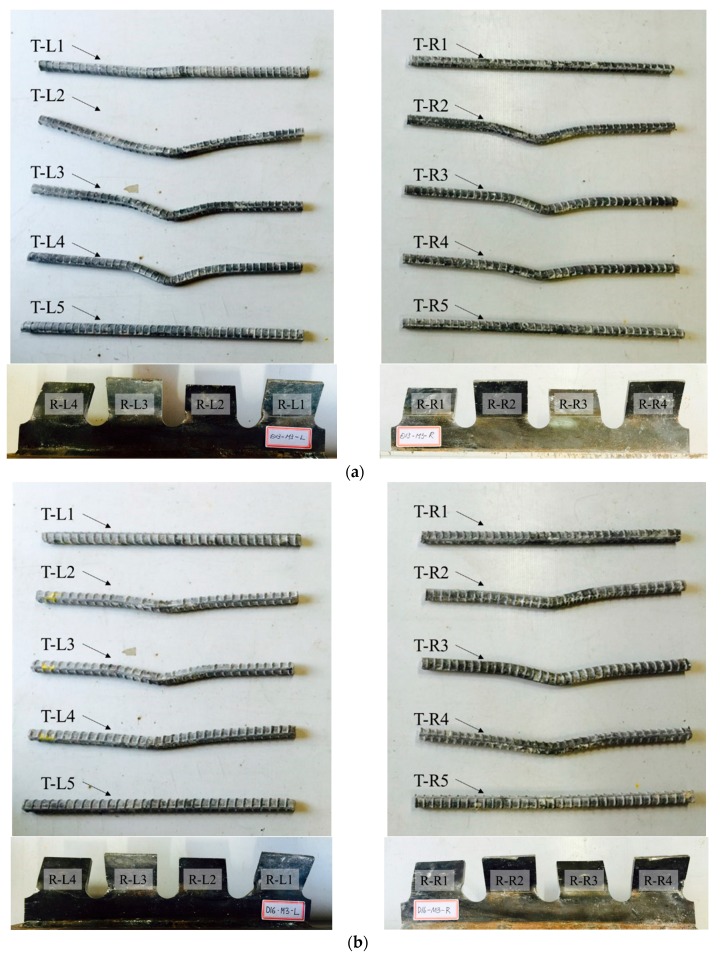
Deformation of transverse rebars and ribs; Stage 5: (**a**) SY-D13-M3; (**b**) SY-D16-M3.

Figure 10, Figure 11 and Figure 12 show the deformations of ribs and transverse rebars for SY-D13-M and SY-D16-M. In the figures, the transverse rebars are labeled as T-L# and T-R#, where “T” refers to the transverse rebar, while “L” and “R” refer to the transverse rebar on the left and right sides, respectively. Furthermore, the group of transverse rebars is numbered from 1 to 5 in order from bottom to top. Similarly, the ribs are labeled as R-L# and R-R#, where “R” refers to the rib, and “L” and “R” refer to the left and right ribs, respectively. The ribs are numbered from 1 to 4 in order from bottom to top.

As shown in Figure 8 and Figure 9, and in Table 7, in the case of the M1 specimen, with approximately 80% shear strength, a slight deformation occurred at the transverse rebar T-R2 of SY-D13-M1, while most other transverse rebars and ribs did not show any significant deformation. However, in the case of SY-D13-M1, multiple transverse rebars (T-L2/L3/L4 and T-R2/R3/R4) and ribs (R-L1; R-R1/R2) showed deformation. These deformations were assumed to be caused by differences in the distances between the ribs and transverse rebars (SY-D13-M: 8.5 mm; SY-D16-M: 7 mm) and the transverse rebar diameter. After local crushing of concrete in the rib hole, the transverse rebars were sheared through increasing shear load, and then the transverse rebars of SY-D16-M with a shorter distance underwent load transfer before those of SY-D13-M. Therefore, the relative slip at shear strength for SY-D16-M is longer than that for SY-D13-M, and the load reduction slope after shear strength of the load–slip curve of SY-D16-M is relatively gradual compared with that of SY-D13-M. Moreover, the relative slips of SY-D13-M2 and SY-D16-M2 are based on the level of stiffness recovery. After stiffness reduction of the load–slip relationship, the strength reduction rate slowly decreased until the strength became constant. In SY-D13-M2, large deformations occurred in several transverse rebars (T-L2/L3 and T-R2), and deformations of several ribs (R-L1 and R-R1) were confirmed. In addition, the degree of deformation was more severe in the transverse rebars than in ribs. As a result, the shear load was transferred to the transverse rebars and ribs, and the shear force was concentrated more on the transverse rebar with a relatively low stiffness than at the rib. SY-D16-M2 showed deformation tendencies similar to SY-D16-M1. In the ultimate limit state of SY-S13-M3, most transverse rebars (T-L2/L3/L4 and T-R2/R3/R4) underwent severe deformation and additional deformation occurred at some ribs (R-L1/L2/L3 and R-R1/R2). In the case of SY-D16-M3, most transverse rebars (T-L2/L3/L4 and T-R2/R3/R4) and some ribs (R-L1/L3 and R-R1/R2) showed deformation. The stubby Y-type perfobond rib shear connectors with transverse rebars (D13 or D16) showed suitable stiffness recovery until the ultimate limit state and did not exhibit brittle failure of the shear connectors owing to sufficient deformation of the transverse rebars and ribs under the ultimate shear load.

## 5. Conclusions

In this study, stubby Y-type perfobond rib shear connectors were proposed for composite frames of building structures by modifying the conventional Y-type perfobond rib shear connectors [32,33,34,35,36]. To evaluate the shear strength and ductility of these connectors, push-out tests of composite structures were conducted using Y-type perfobond rib shear connectors with transverse rebars of different diameters (D13 and D16). The occurrence and propagation of cracks on the surface of concrete slabs during the push-out tests were recorded using a digital camera. After testing, the concrete blocks of the push-out test specimens were destroyed to identify the deformation of the ribs and transverse rebars in each loading stage. The following results were obtained:

(1) The push-out tests conducted using stubby-Y-type perfobond rib shear connectors with different transverse rebars (D13 and D16) indicated that the diameter of the transverse rebars did not considerably affect the change in shear strength. The shear strengths of the stubby Y-type shear connectors with D13 and D16 were 894.6 and 907.4 kN, respectively. That is, their shear strength per unit length (1 m) was approximately 2250 kN/m, which is a significant shear capacity for composite frames of building structures. The experimental results for shear strength showed a difference from the shear strength predicted using the existing equation for Y-type perfobond rib shear connectors (Equation (1); [32]); however, the equation slightly overestimated the influence of the rebar diameter. The increase of the shear strength with respect to the diameter of the transverse rebar was 12.8 (+1.43%) and 279.1 kN (+34.7%) according to the experimental results and the evaluation used in Equation (1), respectively. Based on this result, the term for the diameter of the transverse rebar in Equation (1) evaluates a higher strength than that in the experiment results. Therefore, to verify the applicability of the existing resistance formula, numerous parametric studies are required for stubby Y-type shear connectors.
(2) In terms of ductility, both specimens (SY-D13-M and SY-D16-M) satisfied the ductility standard of Eurocode-4. The ductility of the stubby Y-type perfobond rib shear connector with transverse rebar D16 was 45.1% greater than that with D13. According to the assessment criteria for ductility provided by Kim et al. (2013), the ductility of the stubby Y-type perfobond rib shear connector with transverse rebar D16 was also 28.8% greater than that with D13. These results show that, when stubby Y-type perfobond rib shear connectors with identical rib sizes are used in composite frame structures, the structures with larger-diameter transverse rebars are preferable in terms of ductility.
(3) Concrete crack distributions of the stubby Y-type perfobond rib shear connectors were detected based on the increase in relative slip. Most specimens started to show cracks at the bottom end of the cut rib. The initial cracks in SY-D13-M and SY-D16-M occurred at approximately 75% and 85% shear strength, respectively. In Stage 3, SY-D13-M developed additional vertical cracks, whereas SY-D16-M developed additional horizontal cracks. Then, all the crack patterns of the stubby Y-type perfobond rib shear connector with transverse rebar D13 appeared as pry-out failure of the concrete, while those of the shear connector with transverse rebar D16 displayed overall splitting failure of the concrete. Thus, it can be deduced that the load distribution on the transverse rebar, rib, and concrete is well balanced with increasing transverse rebar stiffness of the shear connector using transverse rebar D16, which has a relatively large cross-sectional area compared with the shear connector with transverse rebar D13. In addition, most rebars exhibited large deformations in Stage 5. These deformations delay concrete crushing in the dowel hole and prevent the brittle failure of shear connections after the ultimate limit state.
(4) The difference of the shear force is low following the diameter of the transverse rebar. However, the size of the rebar affects the ductility and load distribution: a larger size shows better performance. Thus, it is expected that the size of the rebar affects the behavior of the whole shear connector system.

## Figures and Tables

**Table 1:** Results of concrete compressive strength test.

Curing Time	Compressive Strength	
21 days	25.97 MPa	27.17 MPa
	26.33 MPa	
	29.22 MPa	
28 days	28.27 MPa	28.96 MPa
	29.83 MPa	
	28.78 MPa	
Before push-out test	30.08 MPa	29.29 MPa
	28.94 MPa	
	28.84 MPa	

**Table 2:** Results of structural steel tensile strength test.

Specimen	Yield Strength	Tensile Strength	Elongation	Young’s Modulus
S-1	318.48 MPa	422.43 MPa	39%	209 GPa
S-2	338.36 MPa	430.84 MPa	41%	209 GPa
S-3	332.35 MPa	430.75 MPa	41%	209 GPa
S-4	340.73 MPa	440.48 MPa	40%	209 GPa
Average	332.48 MPa	431.12 MPa	41%	209 GPa

**Table 3:** Specifications of the stubby Y-type perfobond rib connectors.

Test Specimen	Y-Shaped Angle	Rib Thickness	Rib Height	Rib Width	Hole Diameter	Transverse Rebar
**SY-D13-M1/M2/M3**	60°	8 mm	50 mm	70 mm	30 mm	D13
**SY-D16-M1/M2/M3**						D16

**Table 4:** List of push-out tests.

Specimen	Terminated Loading Point
SY-D13-M1/SY-D16-M1	At 80% of the shear strength
SY-D13-M2/SY-D16-M2	At the recovered stiffness
SY-D13-M3/SY-D16-M3	At the displacement of 25 mm

**Table 5:** Push-out test results.

Specimen		P_u_ (kN)	δ_uk_ (mm)	δ_u_ (mm)	δ_90_ (mm)	δ_u_/δ_90_
**SY-D13**	M1	925.2	6.61	7.34	1.82	4.03
	M2	904.4	6.20	6.89	1.60	4.31
	M3	898.7	5.85	6.50	1.66	3.92
	Average	894.6	6.90	7.67	1.59	4.82
	Strength predicted using Equation (1) [32]	803.5				
**SY-D16**	M1	904.1	9.55	10.61	2.24	4.74
	M2	907.7	11.20	12.44	1.63	7.63
	M3	939.7	8.78	9.75	2.12	4.64
	Average	907.4	10.01	11.12	1.79	6.21
	Strength predicted using Equation (1) [32]	1082.6				

**Table 6:** Shear resistance evaluated with Equation (1) [26].

Test Specimen	d	h	t	$f_{ck}$	r	$A_{tr}$	$f_{y}$	n	m	s	Q
SY-D13-M1/M2/M3	30	50	8	27	4	126.7	400	3	2	130	803.5
SY-D16-M1/M2/M3	30	50	8	27	4	198.6	400	3	2	130	1082.6

**Table 7:** Crack distribution of stubby Y-type perfobond rib shear connectors.

Stage	SY-D13-M3	SY-D16-M3
**Stage 1**	Initial crack: splitting crack on bottom of concrete	
**Stage 2**	Crack propagation: vertical direction	
**Stage 3**	Additional crack: vertical direction	Additional crack: horizontal direction
**Stage4** **Stage5**	Failure: pry-out	Failure: splitting
